# Optimized Automated Cassette-Based Synthesis of [^68^Ga]Ga-DOTATOC

**DOI:** 10.3390/ph18091274

**Published:** 2025-08-26

**Authors:** Anton Amadeus Hörmann, Johannes Neumann, Samuel Nadeje, Gregor Schweighofer-Zwink, Gundula Rendl, Theresa Jung, Teresa Kiener, Ruben Lechner, Sylvia Friedl, Ursula Huber-Schönauer, Martin Wolkersdorfer, Mohsen Beheshti, Christian Pirich

**Affiliations:** 1 Department of Nuclear Medicine and Endocrinology, University Hospital Salzburg, Paracelsus Medical University, 5020 Salzburg, Austria; 2 Institute of Pharmacy, Department of Pharmaceutical and Medicinal Chemistry and Research and Innovation Center for Regenerative Medicine and Novel Therapies, Strubergasse 21, 5020 Salzburg, Austria; 3 Landesapotheke Salzburg, 5020 Salzburg, Austria

**Keywords:** automated synthesis, DOTATOC, gallium-68, GMP, cassette-based synthesis, neuroendocrine tumors, radiolabeling

## Abstract

**Background:** [^68^Ga]Ga-DOTATOC is widely used in PET imaging of neuroendocrine tumors (NETs) due to its high affinity for somatostatin receptors. Given the short physical half-life of gallium-68 (~68 min), rapid, reproducible, and GMP-compliant synthesis is essential for clinical application. **Methods:** An optimized cassette-based automated synthesis protocol was developed using a commercial cassette. Improvements included direct generator elution into the reactor without pre-purification, use of a SepPak^®^ C18 Plus Light cartridge for purification, replacement of HEPES with 0.3 M sodium acetate buffer (final pH ~3.8), and implementation of a non-vented sterile filter enabling automated pressure-hold integrity testing. **Results:** Across all batches, the synthesis yielded [^68^Ga]Ga-DOTATOC with high radiochemical purity (> 97%) and reproducible decay-corrected radiochemical yields up to 88.3 ± 0.6%. Total synthesis time was approximately 13 min. The final product remained stable for at least 3 h post-synthesis. The use of acetate buffer eliminated the need for HEPES-specific testing, streamlining the workflow. Automated filter testing improved GMP-compliant documentation and reduced radiation exposure for personnel. **Conclusions:** This optimized, cassette-based synthesis protocol enables fast, high-yield, and GMP-compliant production of [^68^Ga]Ga-DOTATOC. It supports clinical theranostic workflows by ensuring product quality, process standardization, and regulatory compliance.

## 1. Introduction

Positron Emission Tomography (PET) has become an essential tool in oncological imaging, particularly for the diagnosis, staging, and therapy monitoring of neuroendocrine tumors (NETs) [1]. Radiolabeled somatostatin analogs such as [^68^Ga]Ga-DOTATOC (DOTA-[Tyr^3^]-Octreotide) are central to this application due to their high specificity for somatostatin receptors, which are commonly overexpressed in NETs [2,3]. The clinical utility of [^68^Ga]Ga-DOTATOC depends on its reliable and GMP-compliant production. Given the short half-life of Gallium-68 (~68 min), rapid and robust synthesis combined with efficient quality control is essential to ensure timely patient administration [4].

Over time, various approaches have been developed for the production of gallium-68 radiopharmaceuticals [5,6,7,8]. Initially, manual syntheses were performed, but were associated with disadvantages such as higher radiation exposure for personnel and potential variability [9]. To improve accessibility and standardization, kit-based methods were developed [10,11]. Kits typically contain all non-radioactive components in pre-aliquoted amounts and allow for simplified radiolabeling, often directly after the elution of gallium-68 from a ^68^Ge/^68^Ga generator. Kit-based syntheses offer advantages such as operational simplicity, lower initial equipment costs, and rapid execution for single-dose applications [12]. Their implementation is feasible even in settings with limited infrastructure, as they often do not require complex synthesis modules. However, residual manual handling steps may result in increased radiation exposure and reduced reproducibility [13,14]. Additionally, fixed kit formulations and production procedures as stated in the SmPC (summary of product characteristics) limit the flexibility to optimize key reaction parameters (e.g., pH, temperature), potentially affecting yield and specific activity.

In contrast, automated synthesis modules—particularly cassette-based systems—have been developed to overcome these limitations. These platforms use single-use, pre-configured cassettes that streamline fluid handling, enhance GMP compliance, and reduce the risk of cross-contamination. Their key strengths lie in process standardization, high reproducibility, and the reduction of operator dose through full automation and shielding. Furthermore, they support precise control of reaction parameters and facilitate the reliable production of higher activities suitable for multi-dose applications [6]. However, they require significant capital investment, incur ongoing costs for disposable components, and demand technical expertise for operation and maintenance. Additionally, low overall synthesis time, in combination with low loss of radioactivity on the cassette, results in high radiochemical yield, which is a crucial parameter for the switch to automated synthesis systems.

Given these advantages and limitations, the choice of an optimal synthesis strategy for [^68^Ga]Ga-DOTATOC in routine clinical use warrants careful consideration.

This study focuses on the synthesis of [^68^Ga]Ga-DOTATOC using two modified approaches based on an in-house-optimized cassette configuration, derived from a commercial cassette and reagent set. Both methods eliminate the need for pre-purification of the gallium-68 eluate (e.g., via cation-exchange), and introduce improvements in synthesis time and radiochemical yield. Additionally, time-efficient quality control procedures, including an automated sterile filter integrity test on the module, were implemented.

The objective is to evaluate both approaches with regard to radiochemical yield, purity, synthesis time, robustness, and operational ease. The findings aim to support radiopharmaceutical laboratories in selecting a suitable synthesis strategy for [^68^Ga]Ga-DOTATOC based on their specific institutional workflows and resources.

## 2. Results

**Figure 1:**
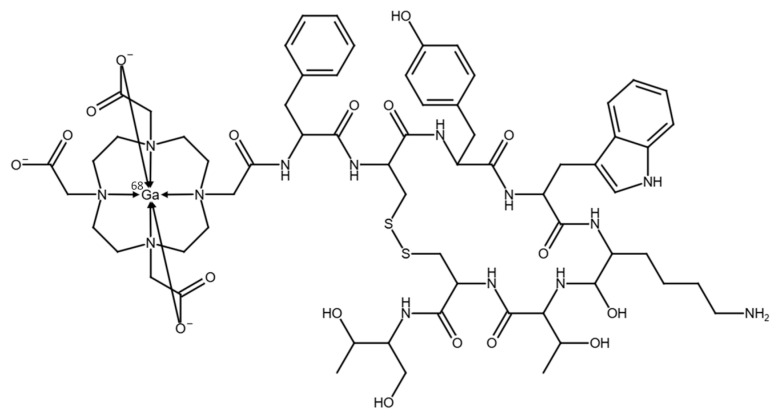
Chemical structure of [^68^Ga]Ga-DOTATOC.

The automated synthesis of [^68^Ga]Ga-DOTATOC (see chemical structure in Figure 1), utilizing the optimized configuration and setup of the synthesis module, was performed in a total synthesis time of approximately 13 min including elution of the generator, radiolabeling, purification, and formulation of the final product. The generators yielded ~65% of the theoretically available gallium-68 activity.

The reference standard [^non-radioactive^Ga]Ga-DOTATOC was successfully labeled, confirmed by a shift in HPLC retention time (R_t_ = 7.30 min) versus DOTATOC alone (R_t_ = 5.50 min; see Figure S1), which was additionally confirmed by electrospray ionization mass spectrometry (ESI-MS) analysis (MW calculated (*m*/*z*) [M+H]^+^: 1489.54; MW found (*m*/*z*) [M+H]^+^: 1489.48). In Figure S2, the ESI-MS spectrum of [^non-radioactive^Ga]Ga-DOTATOC is shown. The identity of [^68^Ga]Ga-DOTATOC was determined by comparing the retention time in the UV-Vis of the cold standard [^non-radioactive^Ga]Ga-DOTATOC with the retention time of the radiodetector signal of [^68^Ga]Ga-DOTATOC (R_t_ = 7.6 min). Both peaks showed similar retention times and were well within the limit of relative retention times (RRTs) of 0.9 and 1.1, confirming the identity of [^68^Ga]Ga-DOTATOC.

### 2.1. Cassette-Based Automated Synthesis of [^68^Ga]Ga-DOTATOC

**Figure 2:**
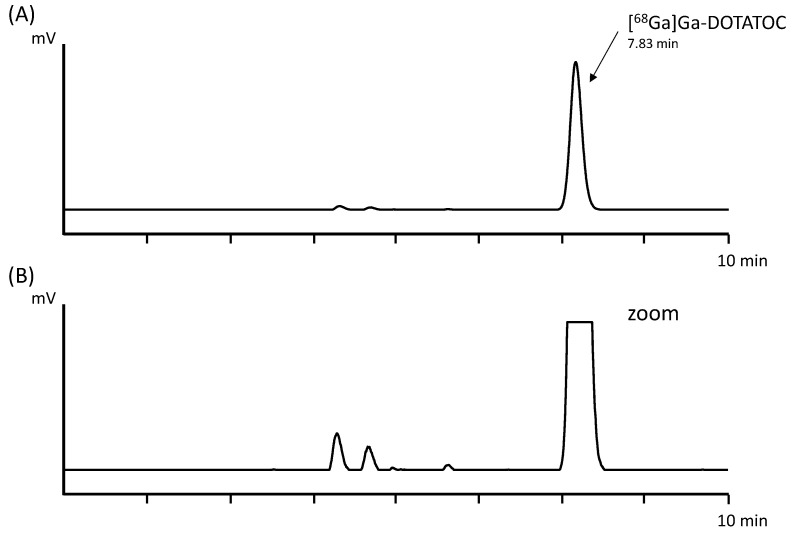
(**A**) Radio-HPLC of [^68^Ga]Ga-DOTATOC using the automated cassette-based radiolabeling method. (**B**) Zoomed chromatogram.

Three batches of [^68^Ga]Ga-DOTATOC were produced using the in-house-developed automated synthesis method with a total activity of 675.2 ± 15.3 MBq of final product, corresponding to an average decay-corrected yield (d.c.y.) of 82.2 ± 2.7% based on the theoretical maximum gallium-68 activity of the generator at that time point. The percentage of the peak corresponding to [^68^Ga]Ga-DOTATOC determined by baseline integration of the radio-HPLC signal was 97.3 ± 0.0%. In Figure 2, an exemplary radio-HPLC chromatogram of the automated cassette-based synthesis is shown.

Table 1 summarizes the results obtained for all batches produced using the automated cassette-based synthesis. Furthermore, Table 2 provides a comprehensive overview of the radiochemical purity, radiochemical yield, and radioactivity distribution for each individual batch.

**Figure 3:**
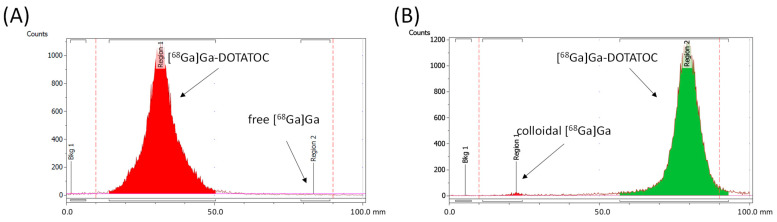
Exemplary iTLC chromatogram of (**A**) [^68^Ga]Ga-DOTATOC using 0.5 M sodium citrate solution, (**B**) 1 M ammonium acetate/methanol mixture from the automated cassette-based synthesis.

The radionuclide incorporation with an R_f_ value of 0.9–1.0 and an R_f_ value of 0.0–0.2, based on radio-iTLC measurements using a 1:1 (*v*/*v*) mixture of 1 M ammonium acetate and methanol, as well as a 0.5 M sodium citrate solution (pH = 5) as the mobile phase, was <1% for both methods. (LOQ = 0.3 kBq/µL). Figure 3 shows exemplary iTLC chromatograms of [^68^Ga]Ga-DOTATOC using automated cassette-based synthesis with both mobile phases.

Across all synthesized batches, the test method for HEPES content, as outlined in the Ph. Eur., consistently demonstrated a significantly lower intensity of the spot with an R_f_ value of approximately 0.2 following staining with iodine vapor. This indicates that the HEPES content in the final product consistently was below the 500 µg/V limit specified in the Ph. Eur. An illustrative iTLC plate, depicting the determination of HEPES content after 4 min of iodine vapor staining, is presented in Figure S3.

### 2.2. Stability Analysis of [^68^Ga]Ga-DOTATOC Using the Automated Cassette-Based Synthesis Method

**Figure 4:**
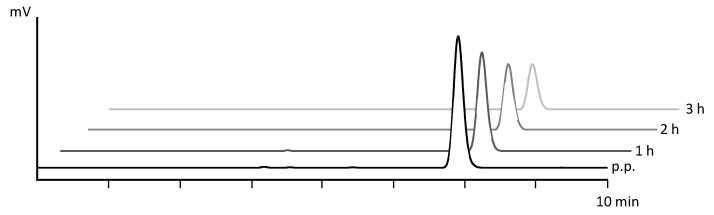
Radio-HPLC chromatograms of [^68^Ga]Ga-DOTATOC over a period of 3 h after synthesis.

The stability of the final product from automated cassette-based synthesis was tested by radio-HPLC and iTLC analysis (*n* = 3) up to 3 h post-preparation (p.p.). The RCP of the preparation was 99.7% ± 0.2% after 3 h p.p., and colloidal gallium-68 species formation did not increase over the subsequent 3 h. Over time, the levels of these by-products also decrease due to radioactive decay, which can cause their signals to fall below the detection limit. The stability data are shown in Table 3, and Figure 4 shows the corresponding exemplary radio-HPLC chromatograms.

### 2.3. Further Optimized Cassette-Based Automated Synthesis of [^68^Ga]Ga-DOTATOC Including Sterile Filter Test

**Figure 5:**
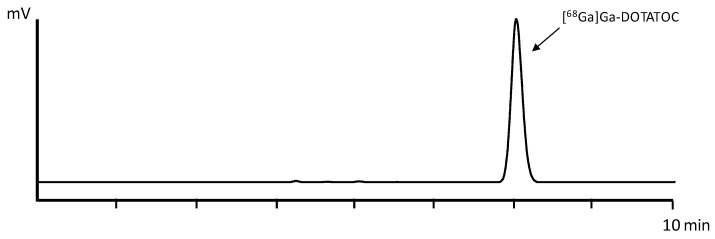
Radio-HPLC chromatogram of [^68^Ga]Ga-DOTATOC using the further optimized automated synthesis method.

Three batches of [^68^Ga]Ga-DOTATOC were synthesized using the further optimized, fully automated cassette-based process, employing sodium acetate as a reaction buffer and a non-vented Millex-GV 0.22 µm sterile filter. The final product yielded a total activity of 665.6 ± 17.4 MBq, corresponding to an average decay-corrected yield of 88.3 ± 0.6%. Radiochemical purity, as determined by baseline integration of the radio-HPLC chromatogram, averaged 97.8 ± 1.3%. An exemplary radio-HPLC profile of the automated synthesis is shown in Figure 5.

Table 4 summarizes the overall performance data for all batches produced using the automated cassette-based synthesis approach. In addition, Table 5 presents a detailed breakdown of the radiochemical purity and decay-corrected yield for each individual batch.

**Figure 6:**
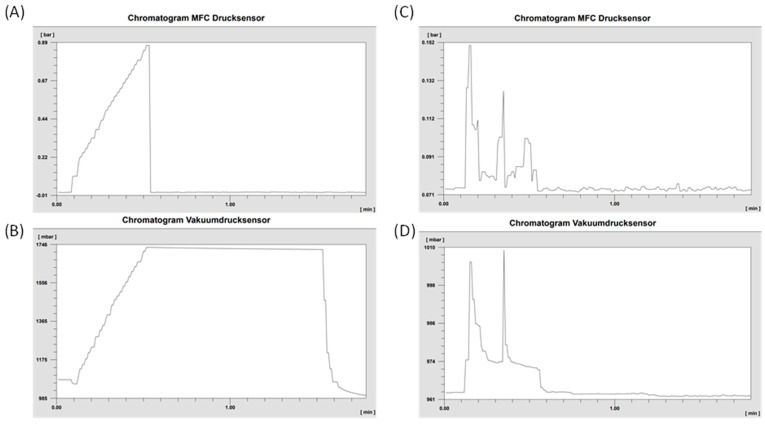
Pressure curves of the intact sterile filter are shown for (**A**) the MFC sensor (nitrogen flow) and (**B**) the VAC sensor (pressure-hold test), along with the corresponding curves for a damaged sterile filter: (**C**) the MFC sensor (nitrogen flow) and (**D**) the VAC sensor (no pressure buildup).

The established pressure-hold test for the sterile filter proved successful across all synthesized batches. Throughout the one-minute observation period, no pressure drop was detected, confirming the integrity of the filters. To further validate the reliability of the system, a broken filter was intentionally tested. As expected, this compromised filter clearly demonstrated an inability to build or maintain pressure. Figure 6 visually presents the distinct pressure curves generated by both the N_2_ flow (measured by the MFC sensor) and the pressure sensor of the vacuum (VAC) sensor, showcasing the difference between an intact and a damaged sterile filter.

## 3. Discussion

[^68^Ga]Ga-DOTATOC has emerged as a key radiopharmaceutical in PET, particularly for imaging NETs. Its clinical relevance is based on its ability to specifically bind to somatostatin receptors (SSTRs), which are overexpressed by many NETs, especially SSTR2 and to a lesser extent SSTR_5_ [2,15]. The high affinity to these receptors enables sensitive visualization of primary tumors and metastases [16,17]. [^68^Ga]Ga-DOTATOC has been used clinically in Europe for over a decade and received Food and Drug Administration (FDA) approval in the United States in 2019, underscoring its established role [18]. Diagnostic utility includes initial staging of NETs, restaging of suspected recurrence, localization of an unknown primary tumor in patients with metastatic NETs, and support in treatment decisions [19]. Numerous studies have shown that PET/CT imaging with [^68^Ga]Ga-DOTATOC is superior to conventional scintigraphy with Indium-111-labeled octreotide (OctreoScan^®^) in terms of sensitivity and spatial resolution [20,21]. Furthermore, [^68^Ga]Ga-DOTATOC plays a key role in theranostic approaches by guiding patient selection and dosimetry for subsequent peptide receptor radionuclide therapy (PRRT) using agents such as [^177^Lu]Lu-DOTATATE [22]. The reliable production of high-quality [^68^Ga]Ga-DOTATOC is essential for accurate imaging and somatostatin receptor quantification. Impaired radiochemical purity can lower image quality, resulting in incorrect patient selection or dose estimations. Automated cassette-based synthesis ensures high reproducibility and product quality, providing reliable diagnostic images to inform therapeutic decisions and maximize PRRT efficacy while preventing unnecessary treatments and side effects [23]. Despite these advantages, the production of [^68^Ga]Ga-DOTATOC poses specific challenges. The short half-life of gallium-68 requires fast and efficient synthesis and quality control processes. Production must also be carried out in compliance with Good Manufacturing Practice (GMP) guidelines to ensure the quality, safety, and efficacy of the radiopharmaceutical for clinical use [5]. A key challenge is to ensure consistently high radiochemical purity and radiochemical yields.

In this study, two optimized cassette-based synthesis protocols were developed and evaluated for their performance in clinical radiopharmaceutical production. A high and consistent radiochemical yield and purity were achieved by radio-HPLC and iTLC analysis. This unequivocally demonstrates robust compliance with all relevant regulatory specifications and specifications of the Ph.Eur [24]. It also highlights the reproducibility of the automated process. The automated cassette system offers several advantages, primarily due to its precise control over critical reaction parameters. This precise control ensures optimal complexation kinetics and minimizes impurity formation, which is particularly beneficial for radiopharmaceuticals with the DOTA chelator [5].

The development and use of a cassette-based synthesis method in this study has achieved optimization through the elimination of a pre-purification step using a strong cation-exchange (SCX) or PS-H+ cartridge and direct elution of the eluate into the reactor. The GalliAd Generator, one of only two generators with marketing authorization, is utilized in this process. At our institution, weekly germanium-68 breakthrough tests are performed to ensure the gallium-68 eluate has the highest purity, which eliminates the need for a dedicated purification step. Furthermore, the generator’s fixed eluate volume of 1.1 mL removes the requirement for eluate concentration. Together, these factors simplify the overall production process, reducing the total synthesis time to approximately 13 min and minimizing potential radioactivity losses [25]. This directly contributes to the higher yields and enhanced efficiency observed. The SepPak^®^ C18 Plus Light cartridge integrated into the automated system effectively performs the necessary purification by retaining the labeled product while allowing the unreacted gallium-68 and other hydrophilic impurities to pass through and holds back colloidal gallium-68 species [26].

The strategic use of the HEPES (4-(2-hydroxyethyl)-1-piperazine ethane sulfonic acid) buffer in automated, cassette-based synthesis is another critical element of the optimized design. This is essential for the efficient and stable radiolabeling of peptides with gallium-68. This study confirmed that the HEPES content in the final product consistently remained below the specified limit of 500 µg/V in the Ph. Eur., ensuring full regulatory compliance and upholding patient safety. The precise volumetric control and buffering capacity facilitated by the automated system are instrumental in achieving consistent product quality. However, the use of this buffer requires additional quality control testing, as it is listed as an impurity in the Ph.Eur and not authorized for intravenous injection [23,24,27,28].

As part of process refinement, two key optimizations were implemented within the cassette-based synthesis protocol: (i) the replacement of HEPES with an acetate buffer and (ii) the substitution of a vented sterile filter with a non-vented configuration.

The substitution of HEPES with a ~0.3 M acetate buffer (final pH ~3.8) offered significant operational and regulatory advantages. In contrast to HEPES, which mandates analytical verification to comply with the Ph. Eur. threshold of ≤500 µg/V, acetate eliminates the need for such batch-specific testing [9]. This adjustment simplifies the release process, reduces time-consuming testing, and preserves high radiochemical purity (97.8 ± 1.3%). Given its established pharmaceutical acceptability, physiological compatibility, and GMP conformity, acetate is a practical and efficient alternative for routine clinical production of ^68^Ga-labeled radiopharmaceuticals.

To further enhance process integrity and enable automated quality control, the vented sterile filter was replaced with a non-vented configuration. This allowed in-line filter integrity testing using a pressure-hold method within the synthesis module. Unlike vented filters, which require manual testing on a separate setup, the non-vented filter enables the capture and archiving of pressure decay data directly in the batch documentation—fulfilling GMP compliance and reducing radiation exposure for staff. Rigorous testing ensures the sterility and safety of the final product by confirming the barrier function of the sterile filter.

The decay-corrected radiochemical yield is crucial as it dictates the number of patients who can undergo a diagnostic procedure. The optimized automated synthesis achieved a decay-corrected yield of 82.2% ± 2.7%, which further improved to 88.3% ± 0.6% with additional optimizations. These yields are notably higher than those reported in the literature for the same synthesis module which are also currently available as a standard method for gallium-68-labeled peptides. For example, Phlak et al. achieved a decay-corrected yield of 72.6% ± 4.9% for [^68^Ga]Ga-FAPI-46 with a total synthesis time of 38 min [29]. In our previous study on the synthesis of [^68^Ga]Ga-FAP-2286, we achieved 71.8% ± 1.9% with a total synthesis time of approximately 35 min [27]. The reduced synthesis time of only 13 min and the higher yield achieved by our optimized automated method allows our clinic to serve more patients per synthesis, leading also to significant cost reductions.

Furthermore, automated synthesis systems inherently support seamless and traceable batch documentation, aligning with stringent GMP requirements for radiopharmaceutical production [5]. However, the described method is currently limited to application with a single GalliAd generator, due to its fixed eluate volume of 1.1 mL and a total reaction volume of 1.9 mL to 2.6 mL. Adaptation to other generator systems or dual-generator setups would require careful re-evaluation of buffer volumes, reactor capacity, and flow parameters.

Taken together, the data clearly support the conclusion that cassette-based automated synthesis represents the method of choice for our routine clinical production of [^68^Ga]Ga-DOTATOC. Given the increasing importance of theranostic approaches and the growing demand for radiolabeled peptides, the transition to automated, standardized production platforms is both a clinical and an operational imperative.

## 4. Materials and Methods

### 4.1. Materials

DOTATOC (Edotreotide) of R&D quality for validation of the automated synthesis and quality control was purchased from MedChemExpress (1 mg, 1 Deer Park Dr, Suite Q, Monmouth Junction, NJ, USA). The peptide was dissolved in 200 µL 96% ethanol and then diluted to 1 mL with 800 µL aqua bidest (Frisenius Kabi AG, Bad Homburg, Germany) to obtain a 1 mg/mL (~0.703 mM) solution. From this solution, 40 µg and a 200 µg fraction was obtained. Materials and reagents for the cassette-based synthesis were provided by SCINTOMICS Molecular, Applied Theranostics Technologies GmbH (Fürstenfeldbruck, Germany).

The successful labeling with the stable isotope of gallium was confirmed by LC-MS using a Shimadzu LC-20 (Shimadzu, Kyoto, Japan) coupled with a Bruker amaZon speed ETD ion trap mass spectrometer (Bruker, Bremen, Germany). For the chromatographic separation a linear gradient ranging from 95:5 water/acetonitrile with 0.1 % formic acid to 5:95 water/acetonitrile over 9 min was used with a flow rate of 0.6 mL/min. MS detection was conducted in ESI positive mode using the UltraScan mode from 300–2000 *m*/*z* with the system tuned to a target mass of 1500 *m*/*z*. For ionization, the dry gas flow was set to 8 L/min with a dry gas temperature of 250 °C and a nebulizer gas pressure of 8.0 psi. Data acquisition and an analysis was performed using Bruker Hystar 4.1 software and Bruker Data Analysis 4.4.

High-performance liquid chromatography (HPLC) mobile phases were purchased as HPLC-quality solvents (Carl Roth GmbH & Co. KG, Karlsruhe, Germany). A 1:1 (*v*/*v*) mixture of 1 M ammonium acetate/methanol as well as a 0.5 M sodium citrate solution, pH = 5, for instant thin-layer chromatography (iTLC) was provided by the hospital pharmacy. The [^68^Ga]GaCl_3_ solution was obtained from a GMP ^68^Ge/^68^Ga generator with a total germanium-68 activity of 1850 MBq at the time of calibration by elution with a 0.1 M HCl solution from the generator (GalliAd, IRE EliT, Fleurus, Belgium). In cooperation with the hospital pharmacy, a GMP-compliant sodium acetate/HCl buffer was developed and purchased for the further optimized synthesis of [^68^Ga]Ga-DOTATOC. To further optimize the automated synthesis, a non-vented Millex-GV 0.22 µm filter (SLGVM33RS, Merck Millipore, Darmstadt, Germany) was used instead of a vented sterile filter.

### 4.2. Optimized Automated Cassette-Based Radiolabeling

**Figure 7:**
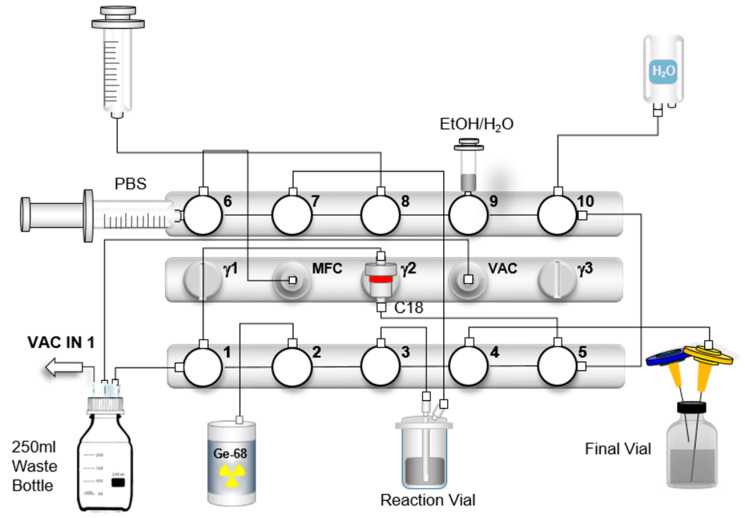
Scheme of the optimized automated cassette-based synthesis of [^68^Ga]Ga-DOTATOC using the GRP-3V synthesis module.

A Scintomics GRP-3V module (Scintomics, Fürstenfeldbruck, Germany) was used for the automated synthesis of [^68^Ga]Ga-DOTATOC in combination with a 2-bench cassette system and a set of reagents and materials. In Figure 7, a scheme of the synthesis is shown.

The following modifications were implemented in comparison to the standard commercial cassette provided by Scintomics: The 50% ethanol vial was replaced by a 5 mL syringe (containing 2 mL of 50% ethanol and 3 mL of air), which was mounted vertically at position 9, replacing the previously connected tubing. Amounts of 11 mL and 2 mL air were withdrawn from the PBS vial connected directly to valve 6 (horizontal); unlike the original setup, its contents were not transferred to the product vial prior to synthesis, but instead used to rinse the C18 cartridge and the sterile filter during final formulation. The generator line was relocated from position 6 to position 2 (both vertical). The spike with the PS-H^+^ cartridge was completely omitted. For nitrogen gas transfer, a line was connected from the Mass Flow Controller (MFC) via position 6 (vertical), using a male–male adapter and a sterile extension tube. In this configuration, generator elution is performed not through the standard 20 mL syringe, but via the module’s integrated vacuum pump, allowing for direct elution into the reaction vial. In addition, the SepPak^®^ C18 Plus Short cartridge was replaced by a SepPak^®^ C18 Plus Light cartridge.

After the cassette was clipped to the module, a “prepare and load” sequence was initiated to close all valves (all valves in position 3) before connecting any reagents. The SepPak^®^ C18 Plus Light cartridge (Waters, Milford, MA, USA) was then preconditioned with 5 mL of 96% ethanol and dried with 10 mL of air before being placed head down on the module, and the cartridge inlet was connected to the line of valve 5 (vertical) and the cartridge outlet was connected to the line of valve 1 (vertical). The final product vial was equipped with a vented filter (combination of a Millex^®^-GS filter and 0.9 × 40 mm cannula) and a vented sterile filter (Cathivex^®^-GV filter and 0.9 × 70 mm cannula). A 5 mL syringe containing 2 mL 50% ethanol and 3 mL air was connected to valve 9 (vertical). A 100 mL water bottle (water for injection) was attached to valve 10 (vertical). The buffer volume required for dissolving the DOTATOC precursor was reduced to 800 μL of 1.5 M HEPES (4-(2-hydroxyethyl)-1-piperazine ethane sulphonic acid) instead of 1.5 mL. The solution was transferred to the reactor vessel. The reactor vessel was then placed in the heating block and connected to the line from valve 3 (vertical, long reactor tube, red) and the line from valve 7 (vertical, short reactor tube, blue). The waste line was connected to valve 1 (horizontal) and the second line from the waste bottle to the vacuum sensor. The 20 mL syringe for liquid transfer was connected to valve 8 (vertical).

Prior to the start of the automated synthesis, a vacuum test is performed to verify that the system is airtight and that the connections are properly tightened to ensure full activity transfer to the module. The pressure decrease is monitored via Scintomics software and the graph is saved as part of the batch documentation. If the pressure decreases below 200 mbar within 1 min, the system is ready for elution of the generator and the system is again vented. Subsequently, all valves are moved to the closed position. Should the system fail to reach a negative pressure below 200 mbar, the synthesis module is not ready for synthesis. This situation requires a systematic check of all connections, the waste bottle, the valve configurations, and the operational status of the vacuum pump. As a last step, the generator line was connected to valve 2 (vertical).

The synthesis started with elution of the generator by turning the knob 90° for 20–30 s. The knob was then returned to the starting position and the synthesis method was started. As a first step, the [^68^Ga]GaCl_3_ eluate was eluted directly into the reactor via valve 3 by vacuum buildup for 2 min and 30 s. After elution of the generator, the valve to the reactor was closed and the vacuum pump switched off. The reactor was then first vented to normal pressure, and then valve 3 and valve 7 were closed to prevent loss of reaction solution by the pressure differential. The vacuum exhaust was then switched to the on position and the entire cassette was vented. The heating block was set to 135 °C. During radiolabeling, the lower bench was rinsed with WFI to remove any free [^68^Ga]GaCl_3_ in the system and the SepPak^®^ C18 Plus Light cartridge was rinsed twice with WFI. After approximately 7 min of radiolabeling, the reaction solution was transferred to the C18 cartridge using nitrogen gas via valve 7 (vertical) to remove any free gallium-68 or colloidal gallium-68 species after radiolabeling. The reactor was then rinsed with 5 mL water and loaded onto the cartridge. After transferring the reaction solution to the cartridge and a subsequent washing step with WFI, the product was eluted with 2 mL of 50% ethanol through a sterile filter into the final product vial and diluted with 11 mL of PBS, and all remaining liquid in the filter was pushed into the final vial via nitrogen gas. Table 6 shows the steps during the automated synthesis.

### 4.3. Further Optimized Automated Cassette-Based Radiolabeling Including Filter Test

**Figure 8:**
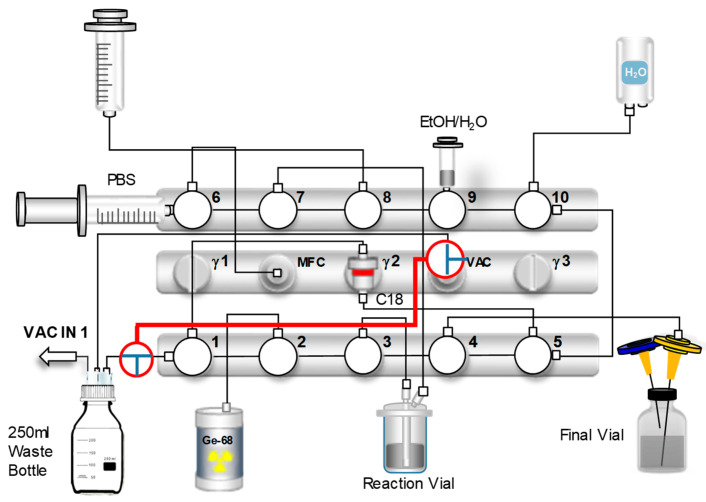
Scheme of the further optimized automated cassette-based synthesis of [^68^Ga]Ga-DOTATOC using the GRP-3V synthesis module including sterile filter testing (setup for synthesis).

To further optimize the entire synthesis and quality control process, an evaluation was carried out regarding the replacement of HEPES buffer with a sodium acetate/HCl buffer. This change aimed to improve reaction conditions and overall product quality, while also eliminating the need for the time-consuming HEPES quantification test using iodine vapor. For this purpose, 1.5 mL of a ~0.3 M sodium acetate solution (pH = 4.4, final reaction pH ~3.8) was used to dissolve the DOTATOC precursor. The resulting solution was then transferred to the reactor. In addition, the heating temperature was reduced from 135 °C to 95 °C. All other synthesis parameters, including the automated steps for generator elution, C18-purification, and formulation, remained consistent, except the final N_2_ flow rate to the sterile filter, with the previously established cassette-based automated synthesis protocol. Upon completion of the synthesis, a critical step to ensure the safety and quality of the final product involved performing a pressure-hold test on the sterile filter. This test required precise manipulation of the nitrogen gas flow within the synthesis module and a pressure buildup. Specifically, two strategically placed 3-way stopcocks were used to allow pressure monitoring via the VAC sensor. For the automated synthesis, one stopcock was on the vacuum sensor (VAC) line and the other on the waste line at valve 1 (horizontal). The scheme of the cassette configuration during the automated synthesis is shown in Figure 8.

After synthesis, the filter (with the needle still attached) was placed onto a separate vented vial for the filter test. To ensure proper pressure for testing, the 3-way stopcock on the VAC sensor was turned 90° clockwise and the other on the waste line 90° counter-clockwise. This effectively isolated the waste bottle, leading to improved pressure buildup. All valves were set to position 3, except valve 4 (position 1) and valve 6 (position 2). With the waste line sealed, the nitrogen gas flow was then rerouted to apply pressure directly to the sterile filter and VAC sensor. The gas flow within the scheme is shown in Figure S4. During the pressure-hold test, compressed nitrogen gas was introduced and the pressure was continuously monitored. Upon reaching ~2 bar, valve 5 was switched to position 2 to maintain system pressure. The pressure drop across the sterile filter was recorded for one minute, after which pressure was equalized by opening the valve connected to the syringe at valve 9 (vertical). Successful maintenance of pressure confirmed filter integrity and the absence of leaks, ensuring protection against microbial contamination of the final [^68^Ga]Ga-DOTATOC product. For validation, the same procedure was conducted with a damaged sterile filter.

### 4.4. Quality Control of [^68^Ga]Ga-DOTATOC

Based on the European Pharmacopoeia monograph “Gallium (^68^Ga) Edotreotide Injection” (2482, Ph. Eur. version 11.3), quality criteria for the final solution of [^68^Ga]Ga-DOTATOC were used for both synthesis methods [24].

For the radio-HPLC analysis of [^68^Ga]Ga-DOTATOC, a Vanquish Core chromatography system (Thermo Fisher Scientific, Waltham, MA, USA) was used, consisting of a quaternary HPLC pump, autosampler, variable UV detector (UV-VIS at λ = 220 nm), and HERM LB 500 NaI radiodetector (Berthold Technologies GmbH & Co, Bad Wildbad, Germany), equipped with an ACE 3 μm C18 column, 150 × 3 mm (ACE-111-1503, Advanced Chromatography Technologies Ltd., Aberdeen, UK). The flow rate was 0.6 mL/min using water/0.1% TFA (solvent A) and acetonitrile/0.1% TFA (solvent B) as mobile phases under isocratic conditions (22% B) for 0–10 min. Chromeleon software (version 7.3.2) was used for data acquisition and analysis. The injection volume was 20 µL.

The identity of [^68^Ga]Ga-DOTATOC was determined by comparing the retention time of the radio-HPLC signal of the radiolabeled product with the UV-Vis retention time of unlabeled DOTATOC and with the stable gallium isotope labeled DOTATOC ([^non-radioactive^Ga]Ga-DOTATOC). The radiochemical purity (RCP) was determined by integrating the peak area corresponding to the main compound, [^68^Ga]Ga-DOTATOC, alongside the peak areas of free gallium-68 and any other unidentified peaks.

To quantify the incorporation of the radionuclide, instant thin-layer chromatography (iTLC) was performed using Agilent iTLC-SG 9 × 1 cm chromatographic plates (Agilent, Santa Clara, CA, USA). Two mobile phases were used: a 1:1 mixture of 1 M ammonium acetate and methanol, and a 0.5 M sodium citrate solution (pH = 5). The percentages of free gallium-68 ions and gallium-68 colloids were quantified using a Scan-RAM radio-TLC scanner equipped with a PS Plastic/PMT detector (LabLogic Systems, Sheffield, UK). The overall radiochemical purity, combining both chromatographic methods, was calculated from the percentage of [^68^Ga]Ga-DOTATOC (Y) determined from radio-HPLC analysis and free or colloidal gallium-68 species (Z) from iTLC using the following Formula (1):

overall RCP % = (100 − Z) × Y(1)

The pH of the final injectable solution was checked using pH indicator strips (Merck, Darmstadt, Germany).

An ISOMED 201 dose calibrator from NUVIA Instruments GmbH (Dresden, Germany) was used to measure the total radioactivity. For half-life measurement for radionuclide identity, the finished product vial was placed in the activimeter and measured for 7 min using embedded software.

Radionuclide identity was verified through gamma-ray spectrometry, and germanium-68 breakthrough was quantitatively analyzed (GabiStar, Raytest, Straubenhardt, Germany) after a minimum decay period of 48 h following release.

To evaluate the HEPES content, a standard stock solution was prepared by dissolving 500 µg of HEPES in water up to the maximum recommended administration volume (V in mL). For the test, a thin-layer plate with silica gel F_254_ was used. The mobile phase consisted of a mixture of methanol, water, and acetonitrile in a ratio of 10:15:75 (*v*/*v*/*v*) and was provided by the hospital pharmacy. Both the test solution, containing the preparation to be examined, and a reference solution, containing a known amount of HEPES, were applied to the plate. Four portions of 2 µL each (maximum dose in mL = 8000 µL/1000) were applied, and after each application, they were dried with a stream of warm air. Development occurred over two-thirds of the plate. After development, the plate was exposed to iodine vapor in a chamber at 30–40 °C for four minutes to visualize the substances. The R_f_ value for HEPES is approximately 0.2–0.3. There is a limit for impurities; the spot from the product solution caused by HEPES must not be stronger than the spot produced by the reference solution.

The bubble point test was chosen for filter integrity testing of the vented filter. For this, after synthesis, the sterile filter was connected to a compressed air line outside the clean room, and the valve was opened. The sterile filter’s needle was immersed in a beaker of water. The accumulating pressure was read from the pressure scale of the valve, and the resulting bubbles in the water were observed.

For the non-vented sterile filter test, an automated pressure-hold test was used.

The stability of the final product was evaluated via radio-HPLC and iTLC analysis for up to 3 h post-production (p.p.) (*n* = 3). The final preparation was stored upright in closed lead shielding at room temperature.

## 5. Conclusions

The optimized cassette-based automated synthesis of [^68^Ga]Ga-DOTATOC enables fast, robust, and GMP-compliant production with high radiochemical yield and purity. Key improvements—including direct generator elution, use of acetate buffer, and automated filter integrity testing—streamlined the process and enhanced reliability. This method is well suited for routine clinical application in theranostic workflows.

## Figures and Tables

**Table 1:** Batch analysis of the automated cassette-based synthesis of [^68^Ga]Ga-DOTATOC. Data are presented as mean ± SD directly after the synthesis.

Parameter	Method	Acceptance Criteria	Results (*n* = 3)
Appearance	Visual inspection	Clear, colorless solution, free of visible particles	conforms
pH	Indicator strip	4.0–8.0	~7
Volume	Graduated vial	13 mL (range 13 ± 1 mL)	conforms
Radionuclide identity	Gamma-ray spectrometry	511 keV; 1022 keV	conforms
Radionuclide identity	Half-life	1.03–1.23 h	1.13 ± 0.00 h
Identity of [^68^Ga]Ga-DOTATOC (comparison with reference)	HPLC	RRT 0.9–1.1	conforms
Radiochemical purity	HPLC	≥95%	97.3 ± 0.0%
Free or colloidal gallium-68 (retardation factor ≤ 0.0–0.1)	iTLC	≤3%	<1%
Free gallium-68 (retardation factor ≥ 0.9–1)	iTLC	≤2%	<1%
Overall radiochemical purity	overall RCP % = (100 − Z) × Y	≥92%	96.3 ± 0.3%
Radiochemical yield	Decay-corrected; calculated	≥70%	82.2 ± 2.7%
Ethanol content	Visual inspection, calculated	Volume > 10 mL	conforms
Filter integrity test	Bubble point test	>3.5 bar	conforms
Radionuclide purity	Gamma-ray spectrometry	Ge-68: ≤0.001% (after ≥48 h)	conforms
HEPES content	Visual; Ph. Eur.	<500 µg/V	conforms

**Table 2:** Single batch analysis of the automated cassette-based synthesis of [^68^Ga]Ga-DOTATOC in terms of radiochemical purity, radiochemical yield, and radioactivity distribution.

Synthesis	1	2	3
RCP (HPLC)	97.2%	97.3%	97.3%
Colloidal gallium-68 species	1.3%	0.7%	0.9%
RCY (d.c.y.)	80.3%	86.0%	80.2%
SepPak^®^ C18 Short Light	8.5%	12.5%	10.4%
Reactor	3.5%	3.7%	8.3%
Sterile filter	7.4%	2.2%	1.7%

**Table 3:** Stability data of [^68^Ga]Ga-DOTATOC up to 3 h after preparation. Data are presented as mean ± SD.

	Acceptance Criteria	p.p.	1 h	2 h	3 h
% RCP (HPLC)	≥95%	97.5 ± 0.3%	98.1 ± 0.4%	98.9 ± 0.4%	99.7 ± 0.2%
% colloidal gallium-68 species	≤3%	≤1%	≤1%	≤1%	≤1%

**Table 4:** Batch analysis of the optimized automated cassette-based synthesis of [^68^Ga]Ga-DOTATOC. Data are presented as mean ± SD directly after the synthesis.

Parameter	Method	Acceptance Criteria	Results (*n* = 3)
Appearance	Visual inspection	Clear, colorless solution, free of visible particles	conforms
pH	Indicator strip	4.0–8.0	~7
Volume	Graduated vial	13 mL (range 13 ± 1 mL)	conforms
Identity of [^68^Ga]Ga-DOTATOC (comparison with reference)	HPLC	RRT 0.9–1.1	conforms
Radiochemical purity	HPLC	≥95%	97.8 ± 1.3%
Free or colloidal gallium-68 (retardation factor ≤ 0.0–0.1)	iTLC	≤3%	<1%
Free gallium-68 (retardation factor ≥ 0.9–1)	iTLC	≤2%	<1%
Overall radiochemical purity	Overall RCP % = (100 − Z) × Y	≥92%	97.1 ± 1.4%
Radiochemical yield	Decay-corrected; calculated	≥70%	88.3 ± 0.6%
Ethanol content	Visual inspection, calculated	Volume > 10 mL	conforms
Filter integrity test	Pressure-hold Test	No pressure drop	conforms

**Table 5:** Single batch analysis of the optimized automated cassette-based synthesis of [^68^Ga]Ga-DOTATOC in terms of radiochemical purity, radiochemical yield, and radioactivity distribution.

Synthesis	1	2	3
RCP (HPLC)	98.1%	99.3%	96.1%
Colloidal gallium-68 species	>1%	>1%	>1%
RCY (d.c.y.)	89.2%	88.0%	87.8%
SepPak^®^ C18 Short Light	8.6%	9.4%	9.16%
Reactor	0.8%	1.4%	1.7%
Sterile filter	1.4%	1.4%	1.4%

**Table 6:** Summary of the steps during automated cassette-based synthesis.

1.	Elution of the generator directly to the reactor using vacuum
2.	Washing the lower bench of the cassette system
3.	Radiolabeling and washing C18 two times with water
4.	Transfer of the reaction solution to the C18 cartridge
5.	Washing of the reactor with water and nitrogen
6.	Washing of the C18 cartridge with water
7.	Elution of the product with 50% ethanol
8.	Washing the C18 cartridge and dilution of the product with 11 mL PBS
9.	Pressing all remaining liquid through the filter using nitrogen flow
10.	Final product and quality control

## Data Availability

The original contributions presented in this study are included in the article/Supplementary Material. Further inquiries can be directed to the corresponding author.

## Supplementary Materials

The following supporting information can be downloaded at https://www.mdpi.com/article/10.3390/ph18091274/s1: Figure S1: UV-Vis HPLC chromatogram of DOTATOC and [^non-radioactive^Ga]Ga-DOTATOC; Figure S2: Electrospray ionization mass spectrometry of [^non-radioactive^Ga]Ga-DOTATOC; Figure S3: Picture of an iodine-stained iTLC for determination of the HEPES content in the final product (R = HEPES reference solution; P = final product); Figure S4: Scheme of the further optimized automated cassette-based synthesis of [^68^Ga]Ga-DOTATOC using the GRP-3V synthesis module with indication of the N_2_ flow (green arrows) for the sterile filter integrity test.
