# Supplementary material for: Optimized Automated Cassette-Based Synthesis of [68Ga]Ga-DOTATOC
Source: Pharmaceuticals (Basel). 2025 Aug 26;18(9):1274. doi: 10.3390/ph18091274 (PMC12472348; doi:10.3390/ph18091274)
Supplement: Supplementary file 1 [file pharmaceuticals-18-01274-s001.zip › pharmaceuticals-3830691-supplementary.pdf]

Optimized Automated Cassette-Based Synthesis of [^{68}Ga]Ga-DOTATOC

Anton Amadeus Hörmann[1,*], Johannes Neumann[2], Samuel Nadeje[1], Gregor Schweighofer-Zwink[1], Gundula Rendl[1], Theresa Jung[1], Teresa Kiener[1], Ruben Lechner[1], Sylvia Friedl[1], Ursula Huber-Schönauer[1], Martin Wolkersdorfer[3], Mohsen Beheshti[1] and Christian Pirich[1]

1 Department of Nuclear Medicine and Endocrinology, University Hospital Salzburg, Paracelsus Medical University, Salzburg, Austria; a.hoermann@salk.at (A.A.H.); sa.nadeje@salk.at (S.N.); g.schweighofer-zwink@salk.at (G.S.-Z.); g.rendl@salk.at (G.R.); t.jung@salk.at (T.J.); t.kiener@salk.at (T.K.); r.lechner@salk.at (R.L.) s.friedl@salk.at (S.F); u.huber-schoenauer@salk.at (U.H-S.); m.beheshti@salk.at (M.B.); c.pirich@salk.at (C.P.)

2 Institute of Pharmacy, Department of Pharmaceutical and Medicinal Chemistry, Paracelsus Medical University Salzburg, 5020, Salzburg, Austria johannes.neumann@stud.pmu.ac.at (J.N.)

3 Landesapotheke Salzburg, Salzburg, Austria; m.wolkersdorfer@salk.at (M.W.)

Table of Contents

Figure S1: UV-Vis HPLC chromatogram of DOTATOC and [$^{\text{non-radioactive}}$Ga]Ga-DOTATOC
Figure S2: Electrospray ionization mass spectrometry of [$^{\text{non-radioactive}}$Ga]Ga-DOTATOC
Figure S3: Picture of an iodine stained iTLC for determination of the HEPES content in the final product (R = HEPES reference solution; P = final product).
Figure S4: Scheme of the further optimized automated cassette-based synthesis of [^{68}Ga]Ga-DOTATOC using the GRP-3V synthesis module with indication of the N_2 flow (green arrows) for the sterile filter integrity test.

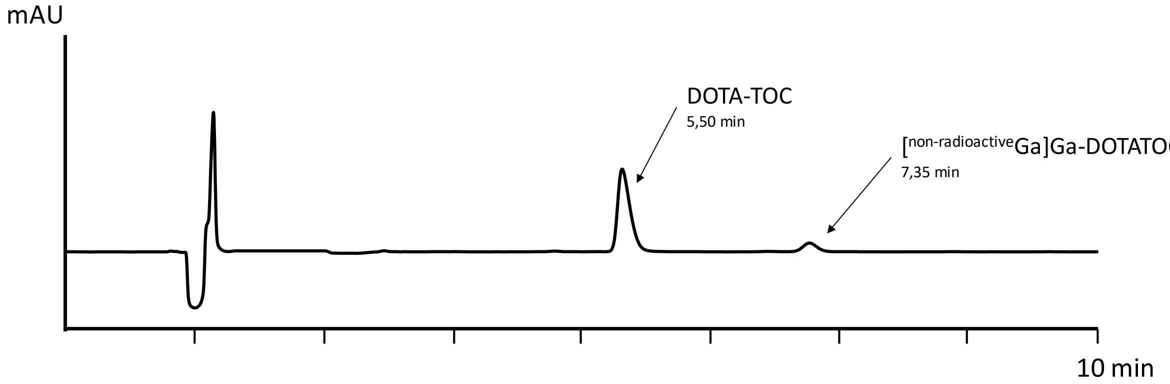

Figure S1. UV-Vis HPLC chromatogram of DOTATOC and [$^{\text{non-radioactive}}$Ga]Ga-DOTATOC

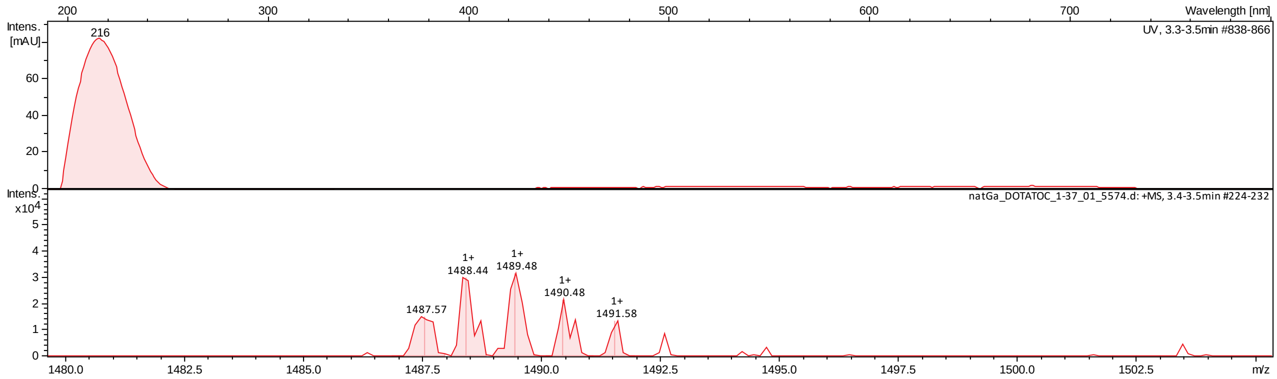

Figure S2. Electrospray ionization mass spectrometry of [$^{\text{non-radioactive}}$Ga]Ga-DOTATOC

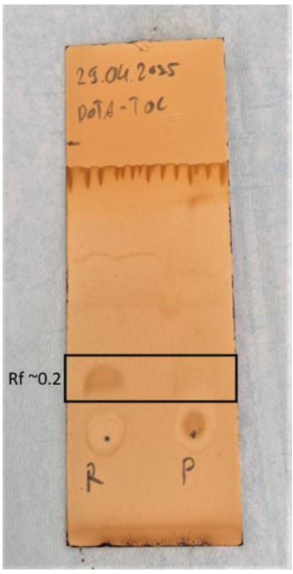

Figure S3. Picture of an iodine stained iTLC for determination of the HEPES content in the final product (R = HEPES reference solution; P = final product).

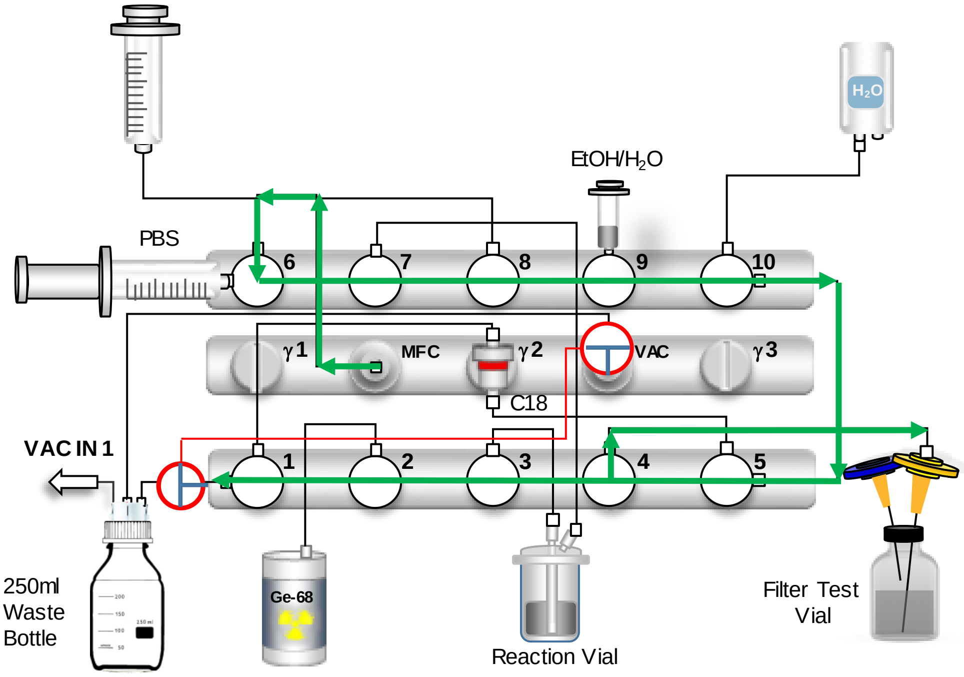

Figure S4. Scheme of the further optimized automated cassette-based synthesis of [^{68}Ga]Ga-DOTATOC using the GRP-3V synthesis module with indication of the N_2 flow (green arrows) for the sterile filter integrity test.
